# Examining the Effectiveness of Web-Based Interventions to Enhance Resilience in Health Care Professionals: Systematic Review

**DOI:** 10.2196/34230

**Published:** 2022-09-06

**Authors:** Catherine Henshall, Edoardo Ostinelli, Jade Harvey, Zoe Davey, Bemigho Aghanenu, Andrea Cipriani, Mary-Jane Attenburrow

**Affiliations:** 1 Oxford School of Nursing and Midwifery Oxford Brookes University Oxford United Kingdom; 2 Warneford Hospital Oxford Health National Health Service Foundation Trust Oxford United Kingdom; 3 Oxford Precision Psychiatry Lab National Institute for Health Research Oxford Health Biomedical Research Centre University of Oxford Oxford United Kingdom; 4 Oxford Institute for Nursing, Midwifery and Allied Health Research Oxford Brookes University Oxford United Kingdom; 5 Department of Psychiatry University of Oxford Oxford United Kingdom

**Keywords:** resilience, health care professionals, depression, psychological stress, internet, mental health

## Abstract

**Background:**

Internationally, the impact of continued exposure to workplace environmental and psychological stressors on health care professionals’ mental health is associated with increased depression, substance misuse, sleep disorders, and posttraumatic stress. This can lead to staff burnout, poor quality health care, and reduced patient safety outcomes. Strategies to improve the psychological health and well-being of health care staff have been highlighted as a critical priority worldwide. The concept of resilience for health care professionals as a tool for negotiating workplace adversity has gained increasing prominence.

**Objective:**

This systematic review aims to examine the effectiveness of web-based interventions to enhance resilience in health care professionals.

**Methods:**

We searched the PubMed, CINAHL, PsycINFO, and Ovid SP databases for relevant records published after 1990 until July 2021. We included studies that focused on internet-delivered interventions aiming at enhancing resilience. Study quality was assessed with the Risk of Bias 2 tool for randomized controlled trial designs and Joanna Briggs Institute critical appraisal tool for other study designs. The protocol was registered on PROSPERO (International Prospective Register of Systematic Reviews; CRD42021253190). PRISMA (Preferred Reporting Items for Systematic Reviews and Meta-Analyses) guidelines were followed.

**Results:**

A total of 8 studies, conducted between 2014 and 2020 and involving 1573 health care workers, were included in the review. In total, 4 randomized controlled trial designs and 4 pre- and postdesign studies were conducted across a range of international settings and health care disciplines. All of these studies aimed to evaluate the impact of web-based interventions on resilience or related symptoms in health care professionals involved in patient-facing care. Interventions included various web-based formats and therapeutic approaches over variable time frames. One randomized controlled trial directly measured resilience, whereas the remaining 3 used proxy measures to measure psychological concepts linked to resilience. Three pretest and posttest studies directly measured resilience, whereas the fourth study used a proxy resilience measure. Owing to the heterogeneity of outcome measures and intervention designs, meta-analysis was not possible, and qualitative data synthesis was undertaken. All studies found that resilience or proxy resilience levels were enhanced in health care workers following the implementation of web-based interventions. The overall risk of bias of all 8 studies was low.

**Conclusions:**

The findings indicate that web-based interventions designed to enhance resilience may be effective in clinical practice settings and have the potential to provide support to frontline staff experiencing prolonged workplace stress across a range of health care professional groups. However, the heterogeneity of included studies means that findings should be interpreted with caution; more web-based interventions need rigorous testing to further develop the evidence base.

**Trial Registration:**

PROSPERO CRD42021253190; https://www.crd.york.ac.uk/prospero/display_record.php?RecordID=253190

## Introduction

### Background

Internationally, “emergency” levels of staff burnout and stress have recently been described and are linked to decreased job satisfaction, absenteeism, and increasing numbers of health care staff leaving their professions [1]. Health care professionals are facing increased pressure to provide high-quality, complex patient care while dealing with staff and infrastructure shortages and chronic, excessive workloads [2-4]. The potential impact of continued exposure to workplace environmental and psychological stressors on the mental health of health care professionals is substantial and is associated with increased depression, substance misuse, sleep disorders, and posttraumatic stress [5]. This picture exists across global health care settings, with staff burnout linked to poor quality health care and reduced patient safety outcomes [6]. The challenges outlined have intensified over the last 18 months owing to the COVID-19 pandemic, with the shock waves initiated undermining the resilience of health systems and the people working within them [7]. Health care professionals have had to support the delivery of expert patient care while rapidly responding to considerable health care challenges, such as understaffing, sickness, personal protective equipment requirements, rapidly changing clinical care policies, and increased patient care demands [8,9]. The psychological impact of delivering health care during COVID-19 has been substantial, with health care professionals working during the pandemic reporting increased levels of stress, distress, anxiety, fear, and depression [10-13]. Rates of burnout among nurses have risen as high as 80% globally during the pandemic [14] and an American study found that physicians’ feelings of burnout reached 61% [15]. As such, the development of strategies to improve the psychological health and well-being of health care staff and mitigate future burnout have been highlighted as key priorities [16,17]. A recent commentary published by the Lancet recommended a series of actions to mitigate this crisis among the health care workforce. These actions included health care practitioners being provided with regular Balint group sessions to discuss clinician-patient relationships with colleagues in comfortable environments, as well as access to resilience training programs for frontline health care staff [18].

The concept of resilience for health care professionals as a tool for negotiating workplace adversity has gained profile over the last decade, with increased importance placed on its benefits [5,19,20]. The term resilience is a dynamic construct that has been framed in several different ways [21]. However, conceptualizing resilience as “coping successfully despite adverse circumstances” recognizes that the tools that health care professionals use to remain resilient are affected by the daily challenges they encounter [22]. The purpose of this review is to measure changes in resilience that relate to relevant psychological constructs such as workplace stress and anxiety. As such, resilience can be defined as an individual’s ability to “adjust to adversity, maintain equilibrium, retain some sense of control over their environment, and continue to move on in a positive manner” [22,23]. Fostering resilience has been highlighted as important in promoting psychological health and well-being, as well as having additional benefits for the recruitment and retention of health care staff [5,22,24]. The protective role of resilience for health care professionals in coping with the ongoing pressures of the COVID-19 pandemic has also been identified [17,25-28].

In England, the National Health Service (NHS) Health and Wellbeing Framework sets the standards for how NHS organizations should support staff to feel well, healthy, and happy at work and advocate for delivering evidence-based staff health and well-being plans [29]. Several interventions have been developed to enhance resilience among health care professionals in both group and individual programs [21,30-36]. Resilience training programs and interventions aimed at health care professionals, such as resilience-building wellness apps [37], have also been developed so that they can be delivered in a range of contexts, including both face-to-face and web-based platforms, and using blended models of delivery [38]. The development of effective, evidence-based digital interventions was identified as playing a potentially important role during the COVID-19 pandemic when the introduction of new infection and prevention control measures constrained the provision of face-to-face interventions within health care organizations and the wider community [39].

A recent Cochrane review of interventions to support the resilience and mental health of frontline health and social care professionals during and after a disease outbreak, epidemic or pandemic, found a lack of evidence to inform the selection of interventions that are beneficial to the resilience and mental health of frontline workers and identified that research to determine the effectiveness of interventions is a high priority. However, the review did not specifically focus on web-based interventions in enhancing resilience among health care professionals [23]. Similarly, a systematic review of interventions aimed at reducing workplace stress in health care workers found limited evidence for reduction in stress levels [40]. Another systematic review found that mindfulness-based stress reduction techniques were associated with improvement in burnout, stress, anxiety, and depression in health care staff [41]. These reviews did not focus specifically on web-based interventions. In addition, another Cochrane review examining the effectiveness of psychological interventions in fostering resilience in health care professionals suggested positive effects of resilience training but low certainty evidence that it resulted in higher levels of resilience and lower levels of depression, stress, or stress perception [38]. None of the reviews focused specifically on web-based training interventions.

### Objectives

This review aimed to assess the effectiveness of web-based interventions in enhancing resilience or reducing anxiety, depression, psychological distress, and trauma in health care professionals. It also seeks to identify whether specific components of web-based interventions effectively enhance resilience, evaluate the acceptability and tolerability of web-based interventions, and assess their potential economic impact. The review included studies dating back to 1990, with an expectation that the findings will be of use in developing mental health interventions for health care professionals during the pandemic.

## Methods

We conducted this systematic review following the recommendations of the PRISMA (Preferred Reporting Items for Systematic Reviews and Meta-Analyses) 2020 statement [42]. The protocol was registered on PROSPERO (International Prospective Register of Systematic Reviews; CRD42021253190).

### Search Strategy

We searched PubMed, CINAHL, PsycINFO, and Ovid SP for published and unpublished evidence on web-based interventions to enhance resilience in health care professionals, with keywords relevant to “internet,” “resilience,” and “health care professionals.” Details of the full search strategy are presented in Textbox 1. We restricted the search to records published between 1990 and July 2021, given that internet interventions did not exist before this year [43]. We inspected relevant reviews and reference lists of the included studies as additional sources of potentially eligible studies for inclusion in the review.

**Search strategy for the systematic review.**

**Full search strategy**

1. PubMed

- (Internet*[tiab] OR online*[tiab] OR “Internet-Based Intervention” [Mesh]) AND (resilien*[tiab] OR coping[tiab] OR cope*[tiab] OR “information processing bias” [tiab] OR adapt*[tiab] OR ruminat*[tiab] OR “Resilience, Psychological” [Mesh]) AND (healthcare worker*[tiab] OR paramedic*[tiab] OR medic*[tiab] OR nurse*[tiab] OR ambulance*[tiab] OR frontline[tiab] OR “Front Line”[tiab] OR “Health Personnel” [Mesh]) AND (“1990/01/01”[Date—Publication]: “3000”[Date—Publication])

2. CINAHL

- AB ([internet* OR online*] AND [resilien* OR coping OR cope* OR adapt* OR ruminat*] AND [healthcare worker* OR paramedic* OR medic* OR nurse* OR ambulance* OR frontline]) AND EM 199001-

3. PsycINFO

- ([Internet* OR online*] and [resilien* OR coping OR cope* OR adapt* OR ruminat*] AND [health care worker* OR paramedic* OR medic* OR nurse* OR ambulance* OR frontline]). ab.
- Limit 1 to yr=“1990-Current”

4. Ovid SP

- ([internet* OR online*] AND [resilien* OR coping OR cope* OR adapt* OR ruminat*] AND [healthcare worker* OR paramedic* OR medic* OR nurse* OR ambulance* OR frontline]). ab.
- Limit 1 to yr=“1990-Current”

### Eligibility Criteria

#### Study Types

We included all primary analytical research studies without limitations regarding study design or publication status. No language or further restrictions were applied to our search strategy.

#### Population

Health care professionals aged ≥18 years were included, regardless of age and sex. Health care professionals were broadly defined as registered personnel directly involved with delivering patient care (eg, nurses, physicians, allied health professionals, and midwives working in any health care setting and clinical specialty).

#### Intervention

Any psychological, behavioral, or educational intervention designed to enhance resilience, with or without an active comparator, was eligible for inclusion. This was because of the limited number of randomized controlled trial studies examining the effectiveness of web-based resilience interventions in the health care setting and the prevalence of studies that used a pre-post test design. We included both fully web-based and partially web-based interventions (eg, mixed web-based and face-to-face delivery or combined web-based and other remote delivery). As resilience is a broad term, interventions include those aimed at enhancing resilience and those aimed at reducing or preventing anxiety, depression, psychological distress, and trauma in the population of interest.

#### Outcome

We investigated the efficacy of web-based interventions in enhancing the resilience in health care professionals. We included any type of outcome measurement or description of resilience and well-being domains that were used as proxy measures of resilience, such as validated and nonvalidated scales of anxiety, depression, well-being, stress, trauma, and posttraumatic stress disorder. As secondary outcomes, we also assessed whether specific components of web-based interventions (eg, length, interactivity, and design features) could enhance resilience in health care professionals, the acceptability and tolerability of interventions, and whether there were any direct or indirect measures of economic impact related to the intervention of interest.

### Study Selection and Data Extraction

Titles and abstracts of the identified records were screened independently by at least 2 members (JH, BA, and ZD) of the review team. The full texts of the potentially eligible studies were subsequently reviewed (JH, BA, MJA, CH, and EO). Any discrepancies were resolved by consensus with a third review team member. Non-English papers were assessed by individuals proficient in that language. Where needed, the original authors were contacted to clarify eligibility and data availability further.

Two review team members (JH and BA) independently extracted study characteristics and outcome data using a digital data extraction form. Any discrepancies were resolved by consensus with a third review team member.

### Risk-of-Bias Assessment

The quality of the included studies was assessed using the Risk of Bias 2 tool for randomized studies [44] and the Joanna Briggs Institute critical appraisal tool [45] for nonrandomized studies.

### Data Synthesis

We conducted a quantitative synthesis by performing a random-effects pairwise meta-analysis. Where not possible, as specified a priori in our study protocol, a qualitative synthesis of the data set was undertaken. Data from the data extraction forms were synthesized and categorized according to the headings in the data extraction table. The qualitative synthesis process followed the recommendations of the Synthesis Without Meta-analysis reporting items PRISMA checklist extension [46].

## Results

### Study Characteristics

**Figure 1:**
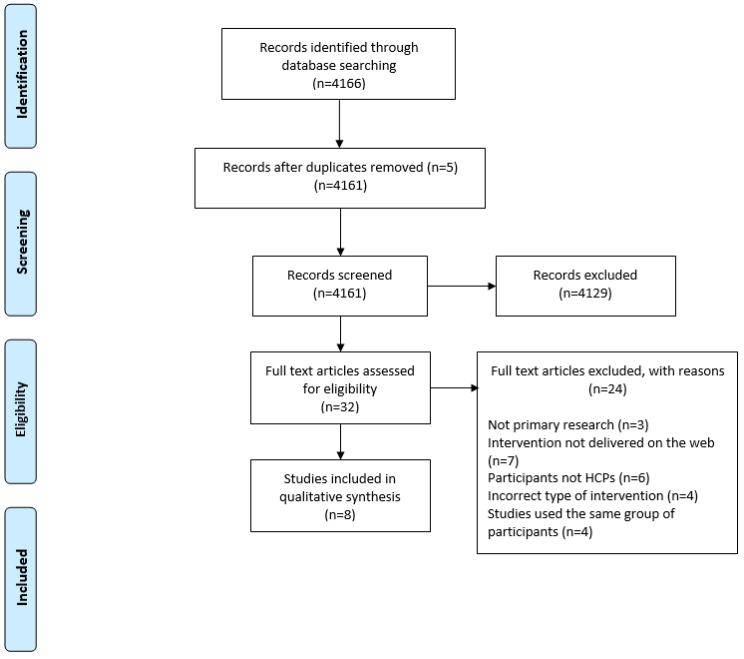
PRISMA diagram outlining screening process used in review.

A total of 4166 papers were identified from the database search, and their titles and abstracts were screened for eligibility based on the inclusion and exclusion criteria. This resulted in 32 remaining studies. The full texts of these studies were screened against the eligibility criteria, leaving 8 studies for inclusion. The screening process is outlined in the PRISMA diagram shown in Figure 1. Of the 8 studies included in this review, all had either randomized controlled trial (n=4, 50%) or pre-post study (n=4, 50%) designs.

Study start dates ranged from 2014 to 2020; one study did not provide this information. The study durations ranged from 1 week to 15 months; one study did not report this information [47]. Studies were conducted across a variety of international settings; 4 were conducted in the United States [48-50], whereas the remainder were conducted in Iran [47], Germany [51], Australia [52], and the Netherlands [53]. Study participants included a range of health care professional disciplines (n=1573), and studies were carried out in academic university settings [49,50,54] or on study programs within health care settings [48,51-53]; one study did not provide this information [47]. Of the 4 health care settings, 1 (25%) study was conducted in a rural primary care setting [52], 1 (25%) was conducted in hospital and ambulance departments [51], 1 (25%) was conducted across 2 urban hospitals and police and fire departments [48], and 1 (25%) was conducted across a variety of health care institutions [51]. The risk of bias for all studies was low, adding to our confidence in the study findings. Characteristics of the included studies are detailed in Table 1.

**Table 1:** Characteristics of studies included in the systematic review.

Study and study title	Study country and setting	Study aim and design	Summary of intervention, analysis, and methods	Participants (n)	Total risk of bias score
Gollwitzer et al [51], 2018; Promoting the self-regulation of stress in health care providers: An internet-based intervention	All over Germany; web-based access	To determine whether health care professionals can downregulate workplace stress using the MCII^a^ tool; randomized controlled trial	Self-regulation by mental contrasting with MCII to reduce stress. Three arms, including control. Surveys; inferential statistics	129	Low
Coifman et al [48], 2021; Boosting positive mood in emergency personnel during the COVID-19 pandemic: preliminary evidence of efficacy, feasibility, and acceptability of a novel online ambulatory intervention	United States; 2 urban hospital centers as well as police and fire departments	To test efficacy of web-based ambulatory intervention aimed at supporting psychological health and well-being of medical personnel and first responders during the COVID-19 pandemic; randomized controlled trial	Daily coping toolkit intervention (low or high dose). Participants undertook 3-6 minutes expressive writing, adaptive emotion regulation or positive emotion generation daily. Surveys; inferential statistics	28	Low
Koppe et al [52], 2016; How effective and acceptable is Web 2.0 Balint group participation for GPs and GP registrars in regional Australia? A pilot study	Australia; rural primary care setting	To evaluate a web-based Balint group for rural physicians and determine effect size for a full-scale trial; pilot randomized controlled trial study	2-hour fortnightly Balint group sessions delivered on the web. Open-ended surveys and thematic analysis; inferential statistics	26	Low
Van der Meer et al [53], 2020; Help in hand after traumatic events: a randomised controlled trial in healthcare professionals on the efficacy, usability, and user satisfaction of a self-help app to reduce trauma-related symptoms	Netherlands; 15 hospitals and 8 ambulance regions	Examining efficacy and evaluating usability and user satisfaction of “SUPPORT Coach” app to reduce trauma-related symptoms; randomized controlled trial	Stand-alone SUPPORT Coach app without use instructions; surveys; inferential statistics	1175	Low
Dehkordi et al [47], 2020; Online Balint groups in health care workers caring for Covid-19 patients in Iran	Iran; virtual	To evaluate the impact of web-based Balint groups on health care workers caring for patients with COVID-19; pre-post study	Web-based Balint group; 1-hour session via Skype 2-3 times a week for 6-8 sessions; surveys; inferential statistics; thematic analysis of free text quantitative data	48-72	Low
Kemper et al [50], 2015; Acute effects of online Mind-Body Skills Training (MBST) on resilience, mindfulness, and empathy	United States; Ohio State University Health Center	To evaluate effect of 1-hour web-based elective MBST for health care professionals on mindfulness, resilience, and empathy; pre-post study	Web-based educational program in MBST: 12×1 hour mind body training modules; 14 hours herbs or dietary supplements; self-reflection surveys; inferential statistics	513	Low
Kopp [49], 2020; Efficacy of mindfulness-based intervention in reducing burnout and increasing resilience in nurses caring for patients with haematologic malignancies	United States; a cancer research institute	To determine feasibility and efficacy of a mindfulness-based intervention program in reducing burnout and increasing resilience in hematology nurses; pre-post study	30-minute web-based mindfulness intervention session; guided 20-minute web-based recording. 5-7 minutes self-guided practice for 1 month; surveys; inferential statistics	40	Low
Hategan and Riddle [54], 2020; Bridging the gap: Responding to resident burn out and restoring well-being	United States; an urban research institution	To promote awareness about wellness and mitigate burn out through learning and building peer support; pre-post pilot study	90-minute web-based resilience curriculum, peer groups, wellness newsletters; survey; thematic analysis	71	Low

^a^MCII: Mental Contrasting with Implementation Intentions.

### Description of Randomized Controlled Trial Study Interventions

Of the 4 randomized controlled trial studies included in the review, all aimed to evaluate the impact of a web-based resilience, or proxy resilience, intervention in health care professionals directly involved in delivering patient care. One study focused on an intervention aimed at health care workers in general [53], one focused on physicians [52], one focused on nurses [51], and one focused on medical personnel and first responders during the COVID-19 pandemic [48]. The duration of the interventions varied, ranging from 1 week to 1 month.

The interventions of the studies were delivered via a variety of formats. These included web-based groups [52], mobile apps [48,53], and other web-based platforms [51]. Some studies adopted specific therapeutic approaches or techniques, including the Balint groups [52], the Mental Contrasting with Implementation Intentions technique [51], and a daily coping toolkit [48]. One study introduced ways to manage emotions and experiences, but the exact content or method implemented was unclear [53].

### Participants

A total of 470 participants were recruited to the randomized controlled trial studies included in the review. However, there was variation in the number of participants recruited to individual studies, ranging from 26 [52] to 287 [53]. The recruited health care workers included nurses, physicians, and first responders.

There was also variance in the proportion of health care workers directly involved in delivering patient care. Three studies involved only health care workers involved in providing direct patient care [51-53]. The fourth study also included participants (31%) who were not directly involved in care delivery, including support staff and health care professionals [48]. One study reported a 100% participant retention rate [48]. The participant completion rates in the remaining studies varied between 64% and 81%.

### Study Outcomes

Differences in outcome measures and intervention designs prevented undertaking a quantitative meta-analysis. One study [53] used an outcome measure, the Resilience Evaluation Scale, to directly measure resilience and found that resilience levels were enhanced in health care workers following implementation of the web-based intervention (Table 2). Three studies used proxy resilience measurement scales to measure the psychological concepts linked to resilience, such as stress, work engagement, professional isolation, and positive outcomes. These included the Burnout Screening Scales II Inventory [51], the Perceived Stress Questionnaire [51], Warr Work-Related Affect Scale [52], the Psychological Medicine Inventory [52], the Professional Isolation Scale [52], posttraumatic stress disorder symptoms using the Posttraumatic Stress Disorder Checklist for Diagnostic and Statistical Manual of Mental Disorders, Fifth Edition [53], and the Peritraumatic Cognitions Inventory [53]. All studies found improvements in these outcome measures after the intervention (Table 2). Some studies used nonstandardized Likert scales to measure specific emotions and concepts such as stress and resilience [48,54], with one study measuring self-rated positive and negative emotion ratings in health care workers [48]. The findings showed that positive emotions significantly increased by 9.4% and negative emotions decreased by 7.8% between the intervention and control groups.

**Table 2:** Outcomes of studies included in the review.

Study	Study title	Study outcome measures	Study results
Coifman et al [48], 2021	Boosting positive mood in emergency personnel during the COVID-19 pandemic: preliminary evidence of efficacy, feasibility, and acceptability of a novel online ambulatory intervention	Daily emotion ratings	Positive emotion ratings showed statistically significant increase in high-dose group compared with low-dose group (mean difference 0.47, SE 0.18). No significant difference in negative emotion ratings between high- and low-dose groups; however, negative emotions decreased more in high compared with low-dose group (mean difference −0.39, SE 0.19).
Gollwitzer et al [51], 2018	Promoting the self-regulation of stress in health care providers: An Internet-based intervention	Overall stress: PSQ-20^a^ and BOSS II^b^; UWES-9^c^	No significant difference in changes to overall stress among control (time point 1: mean 0.16, SD 0.65; time point 2: mean 0.22, SD 0.73), MCII^d^ (time point 1: mean −0.09, SD 0.61; time point 2: mean 0.20, SD 0.63), and IIMCII^e^ (time point 1: mean −0.04, SD 0.41; time point 2: mean 0.05, SD 0.46) groups. No significant differences in UWES-9 scores among control (time point 1: mean 4.06, SD 1.23; time point 2: mean 4.03, SD 1.40), MCII (time point 1: mean 4.22, SD 1.18; time point 2: mean 4.11, SD 1.01), and IIMCII (time point 1: mean 4.43, SD 1.21; time point 2: mean 4.63, SD 1.27) groups.
Koppe et al [52], 2016	How effective and acceptable is Web 2.0 Balint group participation for GPs^f^ and GP registrars in regional Australia? A pilot study	WWAS^g^; PMI^h^; PIS^i^	Significantly higher scores on the WWAS between the intervention (mean 4.09, SD 0.09) and control (mean 3.60, SD 0.12) group; effect size=0.50. Significantly higher scores on PMI scale between the intervention (mean 6.49, SD 0.20) and control (mean 5.43, SD 0.26) group; effect size=0.46. No significant difference on the PIS between the intervention (mean 3.70, SD 0.14) and control (mean 3.63, SD 0.19) group.
Van der Meer et al [53], 2020	Help in hand after traumatic events: a randomised controlled trial in healthcare professionals on the efficacy, usability, and user satisfaction of a self-help app to reduce trauma-related symptoms	RES^j^; SSL-6^k^; Posttraumatic Stress Disorder Checklist for DSM-5^l^ PCL-5^m^; PTCI^n^	RES scores significantly differed; the intervention showed greater increase in RES total scores (psychological resilience; time point 1: mean 24.87, SD 4.67; time point 2: mean 26.54, SD 4.82) compared with control (time point 1: mean 24.88, SD 4.77; time point 2: mean 25.49, SD 5.46). SSL-6 total scores did not differ significantly between the intervention (time point 1: mean 8.38, SD 2.68; time point 2: mean 8.16, SD 2.88) and control (time point 1: mean 8.75, SD 2.95; time point 2: mean 8.16, SD 2.88) groups. No statistically significant differences between intervention (time point 1: mean 10.73, SD 8.17; time point 2: mean 6.08, SD 8.48) and control (time point 1: mean 12.80, SD 12.08; time point 2: mean 8.54, SD 12.74) PCL-5 scores between baseline and follow-up. PTCI total scores significantly differed; intervention showed greater decline in PTCI scores (negative cognitions; time point 1: mean 61.13, SD 23.00; time point 2: mean 49.99, SD 22.78) compared with control (time point 1: mean 63.66, SD 28.66; time point 2: mean 60.83, SD 28.10)
Dehkordi et al [47], 2020	Online Balint groups in healthcare workers caring for Covid-19 patients in Iran	CD-RISC^o^; Corona Disease Anxiety Scale	Significant difference in mean Corona Disease Anxiety Scale score before (mean 35.80, SD 5.09) and after (mean 9.7, SD 2.75) group work. Significant difference pre- (mean 22.80, SD 8.51) and posttest (mean 75.60, SD 6.63) for CD-RISC.
Kemper et al [50], 2015	Acute effects of online Mind-Body Skills Training (MBST) on resilience, mindfulness, and empathy	PSS^p^; BRS^q^; CAMS-R^r^	Significant improvement in PSS scores between the start (mean 17.8, SD 4.9) and end of the module (mean 13.8, SD 6.1). Significant improvement in BRS scores between the start (mean 22.4, SD 4.3) and end of the module (mean 23.3, SD 4.4). Significant improvement in CAMS-R scores between the start (mean 28.0, SD 5.7) and end of the module (mean 29.3, SD 5.2).
Kopp [49], 2020	Efficacy of mindfulness-based intervention in reducing burnout and increasing resilience in nurses caring for patients with haematologic malignancies	CD-RISC; MBI^s^-Health Service Survey	Significant increases in resilience from pretest (mean 28.10) to posttest (mean 30.65), *z*=2.49 (*df*=19). No significant difference in any MBI subscales from pre- to postintervention: emotional exhaustion (3.51 vs 3.23), depersonalization (2.07 vs 2.02), and personal accomplishment (5.06 vs 5.03).
Hategan and Riddle [54], 2020	Bridging the gap: Responding to resident burn out and restoring well-being	Self-rated stress on a 10-point Likert scale	Self-rated stress decreased from 5.5/10 to 2.75/10; this represents a 50% reduction from pre- to postintervention.

^a^PSQ: Perceived Stress Questionnaire.

^b^BOSS II: Burnout Screening Scales II Inventory.

^c^UWES-9: Utrecht Work Engagement Scale.

^d^MCII: Mental Contrasting with Implementation Intentions.

^e^IIMCII: Mental Contrasting with Implementation Intention (that specified when and where participants planned to execute MCII exercises).

^f^GP: general practitioner.

^g^WWAS: Warr Work-Related Affect Scale.

^h^PMI: Psychological Medicine Inventory.

^i^PIS: Professional Isolation Scale.

^j^RES: Resilience Evaluation Scale.

^k^SSL: Social Support List.

^l^DSM-5: Diagnostic and Statistical Manual of Mental Disorders, Fifth Edition.

^m^PCL-5: Posttraumatic Stress Disorder Checklist for DSM-5.

^n^PTCI: Peritraumatic Cognitions Inventory.

^o^CD-RISC: Connor-Davidson-Resilience Scale.

^p^PSS: Perceived Stress Scale.

^q^BRS: Brief Resilience Scale.

^r^CAMS-R: Cognitive and Affective Mindfulness Scale–Revised.

^s^MBI: Maslach Burnout Inventory.

### Description of Pre-Post Test Study Interventions

All pre-post test studies aimed to evaluate the impact of an intervention, delivered either partially or fully on the web, on resilience or related symptoms in health care professionals directly involved in delivering patient care. The duration of interventions generally lasted a few weeks, but one study allowed web-based access to a resilience curriculum throughout an academic year [54].

The interventions in the studies were delivered via a variety of formats including web-based videoconferencing platforms [47], web-based platforms [49], web-based resilience training, peer groups, and wellness newsletters [50,54]. Some studies adopted specific therapeutic approaches or techniques including Balint groups [47] and mindfulness-based interventions [49,50]. One study introduced a way to manage emotions and experiences, but the exact content or method implemented was unclear [54].

Three studies used an outcome measure that directly measured resilience, including the Connor-Davidson Resilience Scale, Brief Resilience Scale, and self-rating of resilience on a visual analog scale [47,50,54]. Other outcomes measured psychological concepts linked to resilience, including the Perceived Stress Scale [50], the Corona Disease Anxiety Scale [47], and Maslach Burnout Inventory [49]. One study used nonstandardized Likert scales to measure specific emotions and concepts such as stress and resilience [54].

### Participants

In total, 1103 participants were recruited to the pre-post studies; however, across studies, this ranged from 10 [47] to 1031 [50]. Two studies focused recruited health care workers in general [47,50], one focused on recruiting physicians only [54], and one focused on recruiting nurses only [49]. Of the 4 studies, 3 (75%) studies included only health care workers involved in providing direct patient care [47,49,54], but 1 (25%) study included participants who were not directly involved in care delivery [50].

The participant completion rate in the pre- and poststudies varied between 50% and 85%. One study involved a web-based module where the completion rate was 50% when defined as the completion of at least a single module; however, this dropped to a completion rate of 4% when considering all the modules [50]. One study did not provide this information [47].

### Study Outcomes

Of the 4 studies, 3 (75%) studies [47,49,50] that directly measured resilience as an outcome measure found that resilience levels were enhanced in health care workers following the implementation of web-based interventions (Table 2). The remaining study used a proxy resilience measurement Likert scale of self-rated stress [54]. The study reported improved psychological well-being for resident physicians, with a postintervention 50% self-reported reduction in stress. However, data analysis included participants who attended in-person groups and had access to web-based resources, with no information reported on the extent of their web-based resource use. Therefore, the extent to which the results were because of the in-person element or the web-based content is unclear. However, one participant commented that “The online resilience curriculum and wellness newsletters were appreciated, and the in-person peer groups were extremely well received.” This suggests that the web-based content was well received, but no further details about its direct benefits were provided [54].

## Discussion

### Principal Findings

The exploratory study findings reported in this review indicate that web-based interventions designed to enhance resilience in health care professionals may be effective in clinical practice settings across a range of health care professional groups. The findings from all included studies showed that web-based interventions significantly improved either resilience or proxy measures of resilience, such as anxiety, depression, well-being, stress, work engagement, or positive emotions. However, the heterogeneity and limited number of randomized controlled trial studies included means that these findings should be interpreted with caution because of a lack of definitive evidence. More randomized controlled trials are needed to produce a robust evidence base on which to develop recommendations related to building resilience among health care professionals. Nonetheless, our review provides a snapshot of the evidence related to this important topical area [18]. The findings may have positive implications regarding the potential of certain types of web-based resilience enhancement interventions in providing support to health care professionals experiencing acute and prolonged stressful conditions in the workplace. This may have long-term benefits in terms of protecting the safe functioning of health systems by preserving the mental health and well-being of staff [7]. The interventions included in this review were tested on health care professionals directly involved in clinical care, demonstrating their potential applicability to clinicians working on the frontline, which warrants further testing in future studies. The included studies were conducted across a range of international settings, ranging from university to hospital, community, urban, and rural environments, and included a wide range of health care professional disciplines, increasing the generalizability of the findings.

The study findings indicate that web-based resilience enhancement interventions may be tolerable and acceptable to a wide range of health care professionals; the importance of resilience enhancement interventions has been cited in recent literature [18]. However, the review findings should be interpreted with caution, with only 50% (4/8) of the included studies having a randomized controlled trial design and 25% (2/8) using nonvalidated outcome measure tools. All interventions were conducted in real-life settings, showing that they are feasible to implement across a variety of health care contexts. In addition, most health care professional participants remained in the study until study completion, with 2 studies having a 100% completion rate, indicating that web-based interventions can be sustained over time and incorporated into the workplace environment. One study included qualitative comments indicating that the web-based components of the intervention were very well received [54]. These findings are important and indicate that web-based interventions can be implemented across health care systems as a valuable, effective, and feasible mechanism for supporting health care professionals to cope with the daily stressors imposed on them. This is especially important in the post–COVID-19 pandemic era, where many face-to-face interventions are impractical, challenging, and pose a potential safety risk. As such, the relevance of web-based training tools and interventions is gaining prominence, and this review provides clear evidence that they can be an important tool for supporting increased resilience in the health care workforce.

Regarding whether specific components of web-based interventions enhance resilience in health care professionals, our findings demonstrated that various formats and therapeutic approaches could effectively improve resilience levels. A range of web-based formats, including videoconferencing, modules, and curricula, were successfully implemented. In addition, a range of intervention techniques, including Balint-style groups, mindfulness, and reflecting on emotions, led to positive changes in resilience or proxy resilience. Although most studies took a purely web-based approach, one was mixed and incorporated additional face-to-face peer group sessions with web-based resilience curricula and wellness newsletters [54]. This suggests that a variety of web-based components can be used to enhance resilience in health care professional groups. However, many of the interventions included interactions with peers or intervention facilitators, suggesting that person-to-person interaction, whether face-to-face or on the web, may increase the likelihood of successful outcomes. This corresponds to previous studies that have demonstrated the benefits of web-based learning [36,55]. The findings point to the benefits of interactive person-to-person features as the key to enhancing intervention acceptability and effectiveness. Future studies should consider ways to incorporate this interactive element within web-based resilience intervention designs to maximize the potential for effectiveness. In addition, consideration should be given to the context within which resilience enhancement interventions are delivered, as health care workers are likely to respond differently when placed under acute versus chronic stressors, as evidenced during the COVID-19 pandemic [56]. The varying durations of interventions included in this review are an indicator of these differing contexts and environments. Thus, interventions that may be effective in acutely stressful environments may have design-different features to interventions that are designed for staff working under chronically stressful conditions; effective interventions offering support for health care workers should account for these differences [56]. None of the studies included in the review measured the economic impact of the intervention within the setting in which it was implemented; therefore, no firm conclusions can be drawn about this. Future research should directly assess the extent to which the implementation of web-based resilience interventions can be cost-effective by considering their long-term impact on staff retention and recruitment, sickness and patient care outcomes, and safety. This aligns with key policy priorities, such as the NHS Long Term Plan and the NHS People Plan, which emphasize that health care staff should be valued, supported to thrive, and treated with respect in the workplace [57,58].

### Limitations

This systematic review has been undertaken rigorously and to a high standard; however, some limitations remain. First, it was not possible to conduct a meta-analysis because of the heterogeneity of the study outcome measurement tools, participant demographics, and study settings. The variation in the characteristics of individual study populations and interventions makes it difficult to draw meaningful comparisons between the included studies, reducing the external validity of the findings. Only 50% (4/8) of the studies included in the review were randomized controlled trial designs. Furthermore, 25% (2/8) of the included studies used nonvalidated scales as outcome measures; this is a potential limitation as it reduces the internal validity of the findings. In addition, 2 studies included a proportion of participants who were not health care workers involved in direct care delivery. For these 2 studies, it was not possible to break down the study findings between direct care and nondirect care staff; therefore, the study findings need to be interpreted with caution as the outcomes could be diluted or exaggerated as a result. Generally, retention rates across the studies were high, demonstrating widespread acceptability of the web-based interventions; however, no data were presented on participants who dropped out of the study and their reasons for this. This information would be helpful to identify any barriers to completion, which could be used to enhance the design features, content, and format of any interventions in the future.

### Comparison With Prior Work

The study findings complement other work in this area that has examined both the effectiveness of resilience enhancement interventions in the health care setting and web-based interventions. Several face-to-face and web-based resilience enhancement interventions for health care professionals have been tested in the workplace environment, with previous systematic reviews finding that they can positively improve psychological well-being [19,23,38,59-63]. McDonald et al [32] successfully developed and implemented a work-based educational intervention to support the development of personal resilience in nurses and midwives in Australia. The intervention led to improvements in colleagues’ levels of honest communication regarding workplace issues, greater respect for each other’s skills and experiences, and a collaborative learning environment, something which is conducive to improved teamwork. It also benefitted participants’ personal and professional lives by enhancing their confidence, self-awareness, assertiveness, and self-care [32]. Henshall et al [21] developed a resilience enhancement program for nurses, consisting of various workshops and tackling areas such as building hardiness, maintaining a positive outlook, achieving work-life balance, reflective and critical thinking, and enabling spirituality. Levels of personal resilience were significantly higher after the program than before the program, with nurses reporting a marked impact on their resilience, self-awareness, confidence, and professional relationships [21].

Many studies have focused on the benefits of interventions in promoting mental health in health care professionals, by reducing depression and anxiety, increasing well-being, and reducing stress, with positive findings. A systematic review exploring interventions to address mental health issues in health care workers during infectious disease outbreaks found that some digital interventions were effective in improving confidence, self-efficacy, anxiety, posttraumatic stress disorder, and ways of coping [61]. Another review found that mindfulness-based interventions had the potential to reduce stress among health care professionals, though the review was not limited to web-based interventions and the quality of the evidence was mixed [59]. A third systematic review to examine the mental health impact of the COVID-19 pandemic on health care workers, and interventions to help them, identified a perceived need and preferences from health care workers for interventions aimed at preventing or reducing negative impacts on mental health. The review included some web-based interventions, but no data on their effectiveness in improving the mental health of participants were collected [60]. These findings reinforce the need for, and potential value of, interventions targeted at health care staff to improve their mental health and promote well-being, something that has been identified in this review.

Despite much literature emphasizing the important benefits of resilience enhancement interventions and web-based learning tools among the health care workforce, no studies to our knowledge have specifically examined the value of web-based resilience enhancement interventions for health care professionals. Our study, therefore, adds to the body of evidence in this field by indicating that web-based resilience interventions can be valuable tools for supporting the psychological well-being of health care professionals working in clinical care settings and can be considered effective, feasible, and acceptable mechanisms for use across a variety of health care settings.

### Conclusions

This review has identified that web-based resilience interventions for health care professionals may be effective tools for enhancing resilience in this population group, are acceptable to the health care workforce, and can be implemented across a range of health care settings and environments. It has been highlighted that a variety of intervention components may be successfully used, but interactive person-to-person features are important design features that should be considered for enhancing success of the intervention. The review findings are important for health care practice as they indicate that simple, yet effective, web-based interventions may play an important role in increasing resilience in the health care workforce. This, in turn, may play a role in protecting health care workers from the pressures and challenges they face in delivering care. Hospital managers, clinicians, and well-being leads should carefully consider using these interventions to enhance resilience and staff well-being in the workplace; however, more web-based interventions need to be tested to enhance confidence in their value and the evidence base. The development of credible resilience enhancement web-based interventions may, in the future, lead to widespread improvements in staff motivation, retention, and recruitment, ultimately improving patient care outcomes.

## Abbreviations

NHS: National Health Service
NIHR: National Institute for Health Research
PRISMA: Preferred Reporting Items for Systematic Reviews and Meta-Analyses
PROSPERO: International Prospective Register of Systematic Reviews
